# Exogenous Abscisic Acid Regulates Distribution of ^13^C and ^15^N and Anthocyanin Synthesis in ‘Red Fuji’ Apple Fruit Under High Nitrogen Supply

**DOI:** 10.3389/fpls.2019.01738

**Published:** 2020-01-24

**Authors:** Fen Wang, Jianchuan Sha, Qian Chen, Xinxiang Xu, Zhanling Zhu, Shunfeng Ge, Yuanmao Jiang

**Affiliations:** State Key Laboratory of Crop Biology, College of Horticulture Science and Engineering, Shandong Agricultural University, Tai’an, China

**Keywords:** apple, abscisic acid, ^13^C, ^15^N, anthocyanin biosynthesis, gene expression

## Abstract

In order to improve the problem of poor coloring caused by high fruit nitrogen in apple production, we studied the effects of different concentrations of abscisic acid (ABA: 0, 50, 100, and 150 mg/L) and fluridone (ABA biosynthesis inhibitor) on the fruit of ‘Red Fuji’ apple (*Malus Domestica* Borkh.) in the late stage of apple development (135 days after blooming) in 2017 and 2018. The effects of these treatments on the distribution of ^13^C and ^15^N and anthocyanin synthesis in fruit were studied. The results showed that the expression levels of ABA synthesis and receptor genes in the peel and flesh were upregulated by exogenous ABA treatment. An appropriate concentration of ABA significantly increased the expression of anthocyanin synthesis genes and transcription factors and increased the content of anthocyanin in the peel. The results of ^13^C and ^15^N double isotope labeling showed that exogenous ABA coordinated the carbon–nitrogen nutrient of apple fruit in the late stage of the development, reduced the accumulation of fruit nitrogen, increased the accumulation of fruit carbon and sugar, provided a substrate for anthocyanin synthesis, or promoted anthocyanin synthesis through the sugar signal regulation mechanism. Comprehensive analysis showed that the application of 100 mg/L ABA effectively improved the problem of poor coloring caused by high fruit nitrogen in the late stage of apple development and is beneficial to the accumulation of carbon in fruit and the formation of color.

## Introduction

As an important agronomic and economic trait, fruit color is an important target trait for breeding and cultivation. Anthocyanin is the main pigment that determines the color of red-skinned apples, made up of cyanidin and glycosyl with glycoside bonds (Ju et al., 1999), which can not only attract consumers but also have antibacterial, anti-inflammatory, antioxidant, anticancer, and cardiovascular disease prevention effects, i.e., beneficial to human health (Zhang et al., 2008; Sun et al., 2013). The content of anthocyanin has become one of the important indicators of phytonutrients or food health. Therefore, how to improve the biosynthesis of anthocyanin has become a hot topic of current research. Due to the one-sided pursuit of high yield and large fruits by fruit farmers, the excessive application of nitrogen fertilizer in apple orchards was more and more common in China, and the amount of nitrogen fertilizer applied far exceeds the demand of the tree (Ge et al., 2018). Most of the fertilizer nitrogen that is not absorbed by the tree is left in the soil profile in an inorganic nitrogen form or organic combination form, and it becomes part of the soil nitrogen pool (Ju, 2014). In the late stage of apple growth and development, high temperature and rainy weather lead to a large amount of organic nitrogen in the root area that is mineralized into inorganic nitrogen that is easily absorbed by trees. This can cause excessive nitrogen in apple fruits. However, this is the key period when the apple tree converts from nitrogen metabolism to carbon metabolism, and high nitrogen in fruits can lead to poor coloring, which affects apple quality and reduces the value of the commodity (Kühn et al., 2011). Therefore, the application of exogenous substances to promote apple coloring under high fruit nitrogen is of great significance to improve the quality of apples.

Anthocyanin is synthesized by the flavonoid pathway and is mainly controlled by two types of genes. The first type is represented by structural genes of the enzymes associated with the encoding synthesis pathway, including phenylalanine ammonia lyase (*PAL*), chalcone synthase (*CHS*), chalcone isomerase (*CHI*), flavanone 3-hydroxylase (*F3H*), dihydroflavonol reductase (*DFR*), and UDP-flavonoid dallyl transferase (*UFGT*). The others are transcription factor genes that regulate the expression of structural genes, including the *MYB* gene and the *bHLH* gene (Honda et al., 2002; Koes et al., 2005; Chopra et al., 2006; Takos et al., 2006; Ban et al., 2007). The content of anthocyanin is closely related to the transcription levels of these genes. For example, studies on *Arabidopsis thaliana* (Peer et al., 2001), carnation (*Dianthus caryophyllus* L.) (Zuker et al., 2002), and grapes (Terrier et al., 2005) found that the expression of the anthocyanin structural gene was positively correlated with the accumulation of anthocyanin. Many studies found that exogenous abscisic acid (ABA) can promote fruit coloring and the biosynthesis of anthocyanin in callus and can induce the upregulated/downregulated expression of maturity-related genes (Kondo et al., 2002; Ruduś et al., 2006; Giribaldi et al., 2010; Li et al., 2014; Ju et al., 2016). Wang et al. (2016) found that exogenous ABA could promote the coloring of citrus fruits, significantly reduce the organic acid content, and affect gene expression and signal transduction pathways involved in sugar and organic acid metabolism, thereby promoting the growth and development of fruit. In sweet cherries and grapes, ABA can affect the expression of structural genes (*DFR*, *LDOX*, and *UFGT*) and *MYB* genes (Gagné et al., 2010; Shen et al., 2014). An et al. (2018) isolated *MdbZIP44* with a yeast screening technique; *MdbZIP44* is an ABA-induced *bZIP* transcription factor in apple and a co-partner with *MdMYB1*. The authors also found that *MdbZIP44* promoted anthocyanin accumulation in response to ABA by enhancing the binding of *MdMYB1* to the promoters of downstream target genes.

Although the molecular mechanism of ABA regulates the synthesis of anthocyanin, the carbon–nitrogen physiological mechanism through which exogenous ABA improves apple coloring is still rarely reported. Therefore, we studied the effects of exogenous ABA on the accumulation and distribution of ^13^C and ^15^N and the anthocyanin synthesis of ‘Fuji’ apple fruit in 2017 and 2018 to provide a scientific basis for improving the poor coloring caused by high nitrogen supply during apple production.

## Materials and Methods

### Experimental Sites and Materials

Field experiments were performed from 2017 to 2018 in an apple orchard at Laishan, Yantai City, Shandong Province, Northeast China (121°43′00″E, 37°50′47″N). The climate is classiﬁed as semi-humid, with an annual average precipitation of 672.5 mm, of which nearly 70% occurs from June to September. The annual mean temperature (1984–2018) is 12.5°C, and there are about 210 frost-free days each year.

Trees were planted in the year 2012 in rows spaced 1.5 m apart with 4 m between the rows and were trained as a slender spindle. The commercially important apple (*Malus* × *domestica* Borkh.) cultivar ‘Red Fuji’ was grafted on the dwarfing interstock M.26 and was then grafted on *Malus hupehensis* Rehd. rootstock (‘Red Fuji’/M.26/*Malus hupehensis* Rehd.). The soil was brown loam with a pH of 5.19, the soil organic matter content was 12.74 g/kg, and NO_3_^−^–N, NH_4_^+^–N, available P, and available K were 40.14, 14.26, 43.47, and 218.57 mg/kg, respectively.

### Experimental Design and Sampling

In the present study, 30 trees with similar growth potential and fruit load were selected and divided into five treatments in 2017 and 2018. The treatments were as follows: treatment 1: CK (0 mg/L ABA, water as control); treatment 2: ABA_50_ (50 mg/L ABA); treatment 3: ABA_100_ (100 mg/L ABA); treatment 4: ABA_150_ (150 mg/L ABA); and treatment 5: Flu (ABA biosynthetic inhibitor, 50 μmol/L fluridone). Treatments were carried out at 135 days after blooming in 2017 and 2018. Treatments were applied to the whole apple fruit evenly with a brush, without drops of water. The treatments were applied three times, once every 4 h. In addition, 0.3 mL/L of Break-Thru^®^ (Evonik Industries, Germany), a non-ionic surfactant, was added to all treatments.

According to isotope labeling, each treatment was divided into two groups with three replicates per group and two trees per replicate as follows: group 1: At the germination stage of apple trees (March 26th), each tree was supplied with 340 g of normal urea (CO(NH_2_)_2_), 210 g of ammonium phosphate ((NH_4_)_2_HPO_4_), and 120 g of potassium sulfate (K_2_SO_4_) as the non-labeled group; group 2: 10 g of ^15^N-urea [CO(^15^NH_2_)_2_, produced by Shanghai Research Institute of Chemical Industry, abundance of 10.22%], 330 g of normal CO(NH_2_)_2_, 210 g of (NH_4_)_2_HPO_4_, and 120 g of K_2_SO_4_ were mixed and applied to the soil beneath each tree. ^13^C pulse labeling was carried out in a labeling chamber at 182 days after blooming in 2017 and 2018. Fertilizer was applied by digging a circular trench with a radius of 30 cm around each tree, with a width and depth of 20 cm. The amount of nitrogen fertilizer applied in each treatment was higher than the recommended amount of nitrogen fertilizer in the orchard, which was 300 kg N/hm^2^ (Drechsel et al., 2015). The growth conditions, cultivation, and management of all treatments were consistent. All the trial plants were subjected to destructive sampling at the fruit maturity stage (185 days after blooming). The fruit was selected from four directions in the middle of the outer part of the crown, with 12 fruits in each replicate. The fruit peel and flesh were immediately frozen in liquid nitrogen and stored at −80°C for further analysis.

### ^13^C Labeling Method

Each plant of group 2 was individually covered and sealed by the labeling chamber, which was composed of 0.1-mm-thick Mylar plastic bags and bracket. The transmittance of sunlight in labeling chamber was 95% of the natural light intensity. One end of a hollow tube was put on a balloon, and the other end had a rubber pipette bulb. According to the inflated state of the balloon, we could determine whether the chamber was well sealed. Put fan and beaker contained with 10 g of Ba^13^CO_3_ (^13^C abundance is 98%) into the labeling room, turned on the fan, and sealed the labeling chamber. Labeling work started at 8:00 a.m. (182 days after blooming). One milliliter of hydrochloric acid (1 mol/L) was injected into the beaker with a syringe every 0.5 h in order to maintain the concentration of CO_2_; ^13^C labeling lasted for 4 h. In order to prevent excessive temperature during labeling process, appropriate amount of ice was added to the bottom of labeling chamber to control the temperature in the range of 28–37°C. All plants were destructively sampled after 72 h (185 days after blooming). The plants of group 1 were destructively sampled and used as a blank for ^13^C labeling (natural abundance of ^13^C).

### Contents of Endogenous Abscisic Acid in the Peel and Flesh

Abscisic acid levels were determined by ultra-high-performance liquid chromatography coupled to tandem mass spectrometry (UHPLC-MS/MS) as described previously (Müller and Munné-Bosch, 2011). In short, 100 mg per sample was extracted with 200 µl methanol/isopropanol/acetic acid 50:49:1 (*v*/*v*/*v*) using ultrasonication and vortexing (Branson 2510 ultrasonic cleaner, Bransonic, Danbury, CT, USA) for 30 min. Deuterium-labeled ABA was then added, and after centrifugation at 600×*g* for 15 min at 4°C, the pellet was re-extracted using the same procedure. Supernatants were pooled and filtered through a 0.22-µm polytetrafluoroethylene (PTFE) filter (Waters, Milford, MA, USA) before analyses. ABA levels were analyzed using UHPLC-ESI-MS/MS as described in Müller and Munné-Bosch (2011). Quantification was performed considering recovery rates for each sample by using a deuterium-labeled internal standard. Three replicates were conducted for each treatment.

### Anthocyanin Content in the Peel

The total anthocyanin content of the apple peel was measured according to Xie et al. (2013), with slight modifications. The apple peel was collected by a hole-punch with a 0.65-cm radius and was extracted with 1% (*v*/*v*) HCl–methanol for 16 h at room temperature in the dark. After centrifugation at 8,000×*g* for 15 min, the absorbance of supernatants was measured at 530, 620, and 650 nm with a spectrophotometer. The content of anthocyanin was expressed as nanomoles of cyanidin-3-galactoside in 1 cm^2^ of fresh sample using a molar extinction coefficient of 3.43 × 10^4^ (Ubi et al., 2006). Three replicates were conducted for each treatment.

### Contents of ^15^N and ^13^C

All the trial plants were subjected to destructive sampling, and the whole plant samples were divided into leaves, annual branches, perennial branches, trunk, roots, and fruits. The samples were washed by clear water, detergent, clear water, and 1% hydrochloric acid in order, and then with deionized water three times. The samples were then dried at 80°C, followed with homogenization using an electric grinder and filtration with a 0.25-mm mesh screen. The samples of group 2 were used to determine the content of nitrogen and the abundance of ^15^N and ^13^C, and those of group 1 were used to determine the natural abundance of ^13^C as a blank control of the corresponding organs of group 2. The content of nitrogen was determined by the Kjeldahl method, and the abundance of ^15^N was measured with a ZHT-03 mass spectrometer made in the Beijing Analytical Instrument Factory (Chinese Academy of Agricultural Sciences). The abundance of ^13^C was measured with a DELTAV^plus^XP advantage isotope ratio mass spectrometer and analyzed by the China Academy of Forestry Sciences Stable Isotope Laboratory. Three replicates were conducted for each treatment.

**Calculation of ^15^N**

Ndff(%)=abundance of N15 in plant–natural abundance of N15abundance of N15 in fertilizer–natural abundance of N15×100%

N15 distribution rate (%)=N15 absorbed by each organ from fertilizer(g) total N15 absorbed by plant from fertilizer(g)×100%

**Calculation of ^13^C**

Abundance of ^13^C:Fi(%)=(δC13+1,000)×RPBD(δC13+1,000)×RPBD+1,000×100%

*R*_PBD_ (standard ratio of carbon isotope) = 0.0112372

Carbon content of each organ: C*_i_* = amount of dry matter (g) × total carbon content (%).

Content of ^13^C of each organ: Ci13(mg)=Ci×(Fi−Fnl)100×1,000

F_nl_: no ^13^C labeling, natural abundance of ^13^C of each organ.

^13^C distribution rate: C13(%)=Ci13Cnet absorption13×100%.

### Soluble Sugar Content

The content of soluble sugar in fruit was measured using anthrone colorimetry (Liu et al., 2018). Samples were put into a test tube, to which 5 ml distilled water was added and mixed after cutting the samples into pieces. After 30 min boiling water bath, the supernatant was collected. This step was repeated twice, and the volume of the solution was adjusted to 10 ml using distilled water. The absorbance of the solution was determined at 630-nm wavelength after adding sulfuric acid and anthrone. Three replicates were conducted for each treatment.

### RNA Extraction and Gene Expression Analysis

The total RNAs of the fruit peel and flesh were isolated with the cetyltrimethylammonium bromide (CTAB) method. First-strand cDNA synthesis was performed using the TransScript^®^ One-Step gDNA Removal and cDNA Synthesis Supermix (Transgen Biotech, China). The cDNA was diluted tenfold and 1 μl of the diluted cDNA was used for quantitative reverse transcription-polymerase chain reaction (qRT-PCR) analysis. The reaction was carried out using chamQ SYBR qPCR Master Mix (Vazyme, China) as described by the manufacturer. The reaction procedure was as follows: pre-denaturation at 95°C for 30 s, followed by 45 cycles of denaturation at 95°C for 5 s, annealing at 58°C for 10 s, extension at 72.0°C for 30 s, incubation at 65°C for 20 s, dissolution from 55°C to 95°C, increasing by 0.5°C/s, and finally, termination of the reaction. Relative gene expression of mRNA was calculated using the 2^−ΔΔCT^ method (Livak and Schmittgen, 2001). All qRT-PCR primers are shown in **Table 1**, and *MdACTIN* was used as the reference gene. Three technical replicates and three biological replicates were prepared for each treatment.

**Table 1:** Primer sequences for qRT-PCR.

Gene name	Forward sequence of the primers (5′→3′)	Reverse sequence of the primers (5′→3′)
*MdACTIN*	TGACCGAATGAGCAAGGAAATTACT	TACTCAGCTTTGGCAATCCACATC
*MdNCED*	GACGACGGTTATATTCTG	GTAGCCTCCAACTTCATA
*MdPYR*	AATGAGGCACCCGTTATG	GTCACAATCAGGCACTTCT
*MdCHS*	GGAGACAACTGGAGAAGGACTGGAA	CGACATTGATACTGGTGTCTTCA
*MdCHI*	GGGATAACCTCGCGGCCAAA	GCATCCATGCCGGAAGCTACAA
*MdF3H*	TGGAAGCTTGTGAGGACTGGGGT	CTCCTCCGATGGCAAATCAAAGA
*MdDFR*	GATAGGGTTTGAGTTCAAGTA	TCTCCTCAGCAGCCTCAGTTTTCT
*MdUFGT*	CCACCGCCCTTCCAAACACTCT	CACCCTTATGTTACGCGGCATGT
*MdMYB1*	TGCCTGGACTCGAGAGGAAGACA	CCTGTTTCCCAAAAGCCTGTGAA
*MdbZIP44*	AGCAGCACCTGGACGATCTGACG	GGTGAAGCATGTCGGCAGTGGCC

### Statistical Analysis

Origin 8.0 (OriginLab Corporation, Northhampton, MA, USA) was used for figure drawing. Data were analyzed with SPSS 17.0 (SPSS, Inc., Chicago, IL, USA) by using one-way factorial analysis of variance (ANOVA). In all cases, differences were considered significant at a probability level of *P* ≤ 0.05. Furthermore, correlation analyses using Pearson’s correlation were performed.

## Results

### Endogenous ABA Content and ABA-Related Gene Expression in the Peel and Flesh

The contents of endogenous ABA in the peel of CK and ABA treatments were higher than that in the flesh, while the opposite was observed in the Flu treatment (**Table 2**). After exogenous ABA treatment, the ABA content in the peel and flesh was higher than that of control and increased with the increase in exogenous ABA concentration. In 2017 and 2018, the ABA content of the peel was increased by 13.00–43.23% and 12.64–42.02%, respectively, compared with the control, while the ABA content of the flesh was increased by 13.41−57.65% and 12.78−55.51%, respectively. The endogenous ABA content in the peel and flesh of the Flu treatment was lower than that in the control. The results showed that exogenous ABA treatment promoted the synthesis of endogenous ABA in the peel and flesh and increased the content of endogenous ABA in fruit (**Table 2**).

**Table 2:** Effects of different treatments on ABA and anthocyanin contents in apple fruit in 2017 and 2018.

Year	Treatment	Endogenous ABA content (ng/g FW)		Anthocyanin content in the peel (nmol/cm^2^)
		Peel	Flesh	
2017	CK	314.03 ± 13.45c	272.86 ± 11.73d	10.08 ± 0.78b
	ABA_50_	354.85 ± 15.20b	309.46 ± 13.25c	11.58 ± 0.55a
	ABA_100_	417.26 ± 21.68a	369.64 ± 26.74b	12.97 ± 1.00a
	ABA_150_	449.78 ± 25.17a	430.15 ± 16.93a	11.74 ± 0.91a
	Flu	207.99 ± 11.70d	225.16 ± 21.44e	8.04 ± 0.49c
2018	CK	332.78 ± 13.85c	290.91 ± 12.08d	10.14 ± 0.82c
	ABA_50_	374.83 ± 15.65b	328.08 ± 13.65c	11.72 ± 0.58b
	ABA_100_	439.11 ± 22.33a	390.06 ± 27.54b	14.19 ± 1.05a
	ABA_150_	472.60 ± 25.93a	452.39 ± 17.43a	11.89 ± 0.96b
	Flu	223.56 ± 12.05d	241.25 ± 22.08e	7.99 ± 0.52d

Data are presented as the mean ± SD of three replicates. Different letters within a column indicate statistically significant differences between the means (P < 0.05).

**Figure 1:**
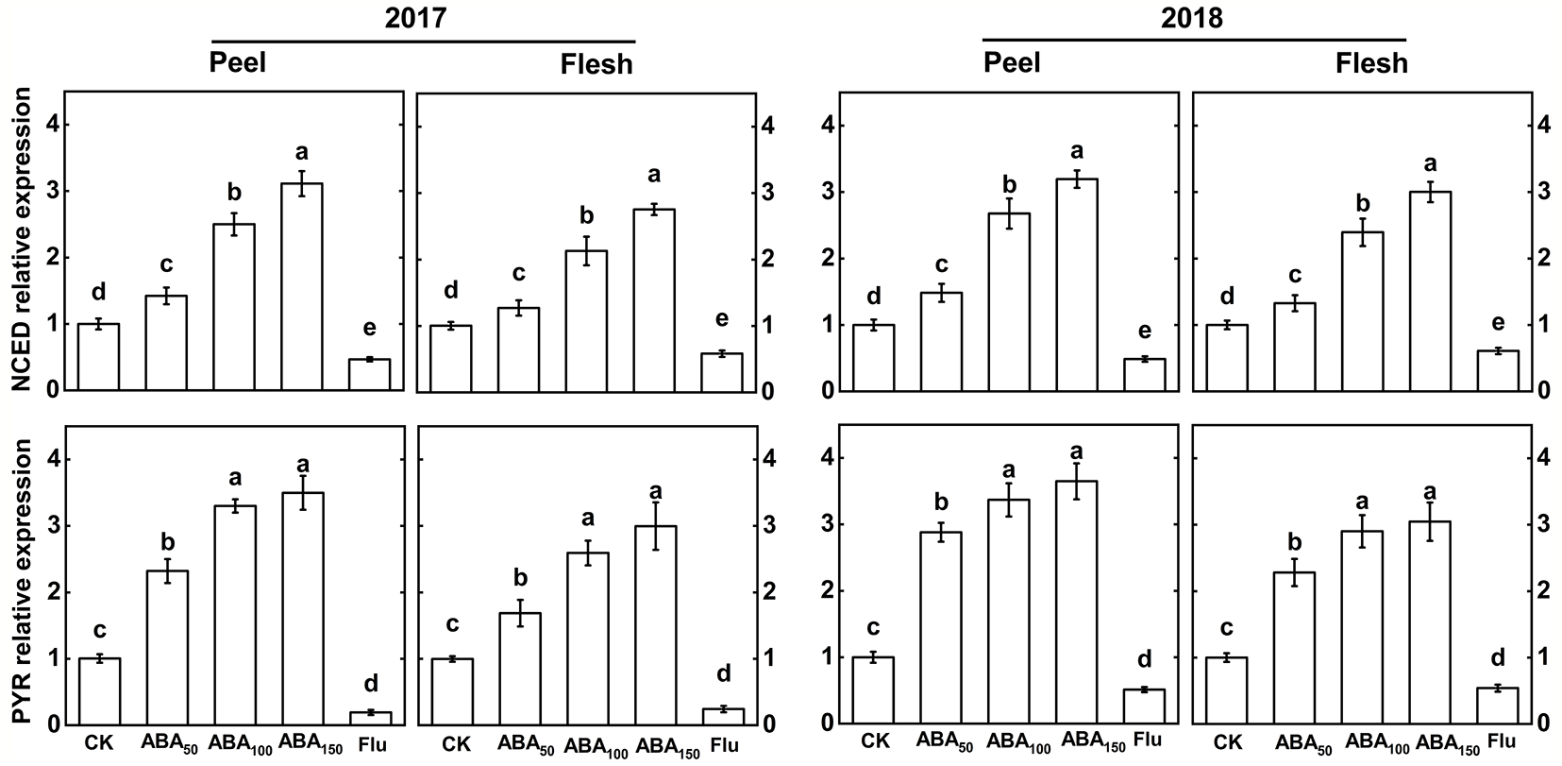
Effects of different treatments on the key gene expression related to ABA in the fruit peel and flesh in 2017 and 2018. The *vertical bar* indicates the standard deviation of three replications. *Different letters* indicate statistically significant differences (*P* < 0.05).

The difference in ABA synthesis gene and receptor gene expression between apple peel and flesh was analyzed by quantitative real-time PCR (**Figure 1**). In 2017 and 2018, the relative expression levels of the ABA synthesis gene (*MdNCED*) and receptor gene (*MdPYR*) in the peel and flesh increased with an increase in the exogenous ABA concentration, and the highest levels were reached in the 150-mg/L ABA treatment. Compared with the control, the Flu-treated gene expression was downregulated (**Figure 1**).

### Anthocyanin Content and Anthocyanin Biosynthesis-Related Gene Expression in the Peel

The anthocyanin content in the peel increased first and then decreased with the increase in exogenous ABA concentration in both years (**Table 2**). The anthocyanin content in the 100-mg/L ABA treatment reached the highest level, which was 28.67% and 39.94% higher than that in the control in 2017 and 2018, respectively, whereas the anthocyanin content in the peel of the fluridone treatment was significantly lower than that of the control. The results showed that exogenous ABA promoted the synthesis of anthocyanin in the peel (**Table 2**).

**Figure 2:**
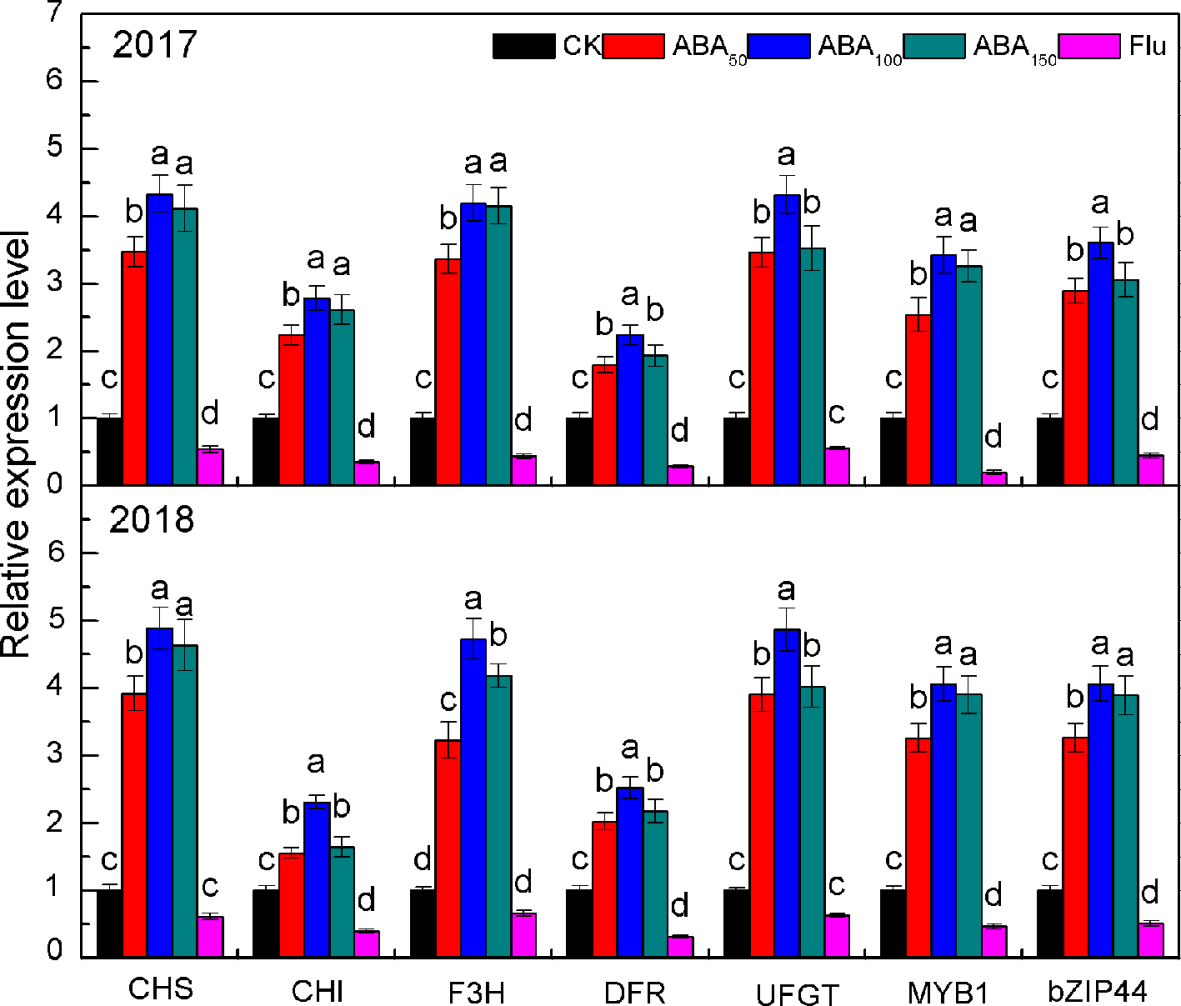
Effects of different treatments on key gene expression related to anthocyanin biosynthesis in the peel in 2017 and 2018. The *vertical bar* indicates the standard deviation of three replications. *Different letters* indicate statistically significant differences (*P* < 0.05).

As presented in **Figure 2**, the expression of anthocyanin synthesis genes (*MdCHS*, *MdCHI*, *MdF3H*, *MdDFR*, and *MdUFGT*) and transcription factors (*MdMYB1* and *MdbZIP44*) increased first and then decreased with the increase in exogenous ABA concentration and reached the highest level at 100 mg/L ABA in both years, among which *MdCHS*, *MdF3H*, *MdUFGT*, *MdMYB1*, and *MdbZIP44* increased the most. By contrast, the expression levels of all genes in the Flu treatment were downregulated compared with the control (**Figure 2**).

### The ^13^C Distribution Rate

**Figure 3:**
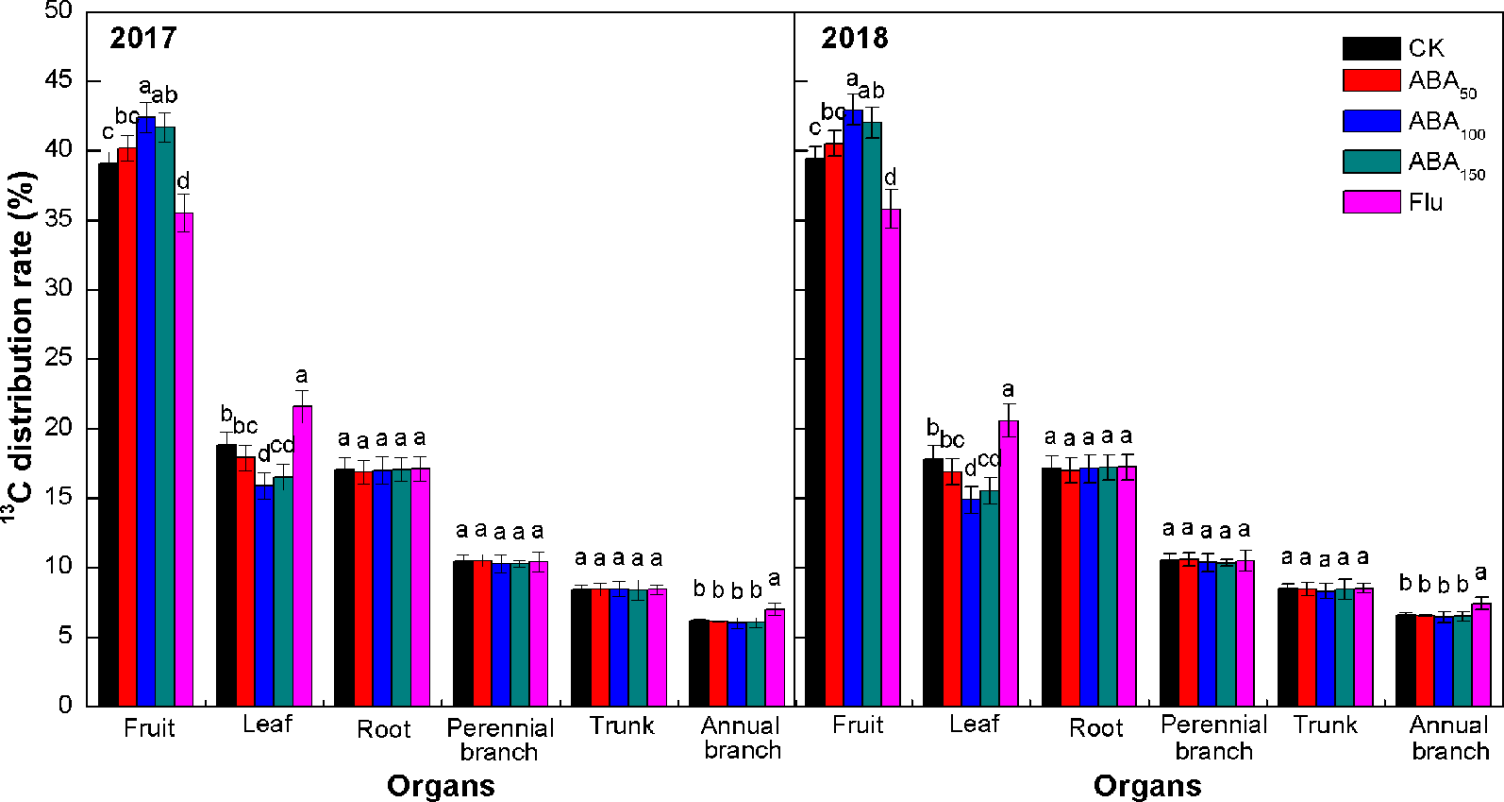
Effects of different treatments on the ^13^C distribution rate at the fruit maturity stage in 2017 and 2018 (^13^C distribution rate refers to the ratio of the ^13^C content of each organ to the amount of net ^13^C absorbed by the plant). The *vertical bar* indicates the standard deviation of three replications. *Different letters* indicate statistically significant differences (*P* < 0.05).

The proportion of ^13^C assimilates assigned to each organ is related to its competitive ability, which referred to the ability of absorbing ^13^C from the labeled leaves in the active parts of metabolism and growth. The ^13^C distribution rates for each treatment were consistent in both years, among which the fruits had the highest values followed by the leaves, roots, perennial branches, trunk, and annual branches (**Figure 3**). Exogenous ABA treatment increased the ^13^C distribution rate of fruits, which increased first and then decreased with the increase in the ABA concentration. The ^13^C distribution rate of fruits treated with 100 mg/L ABA was the highest and increased by 8.53% and 9.04% compared with the control in 2017 and 2018, respectively. Exogenous ABA treatment also reduced the ^13^C distribution rate of leaves and annual branches to varying degrees. The ^13^C distribution rate of leaves and annual branches treated with 100 mg/L ABA was the lowest. Compared with the control, fluridone treatment significantly reduced the ^13^C distribution rate of fruits and significantly increased the ^13^C distribution rate of leaves and annual branches. No treatment had a significant effect on the ^13^C distribution rate of storage organs (roots, perennial branches, and trunk). The results showed that ABA treatment improved the competitiveness of fruit with respect to ^13^C and improved the transport of ^13^C from vegetative organs (leaves and annual branches) to fruit.

### The Ndff Values and ^15^N Distribution Rate of Plant Organs

**Figure 4:**
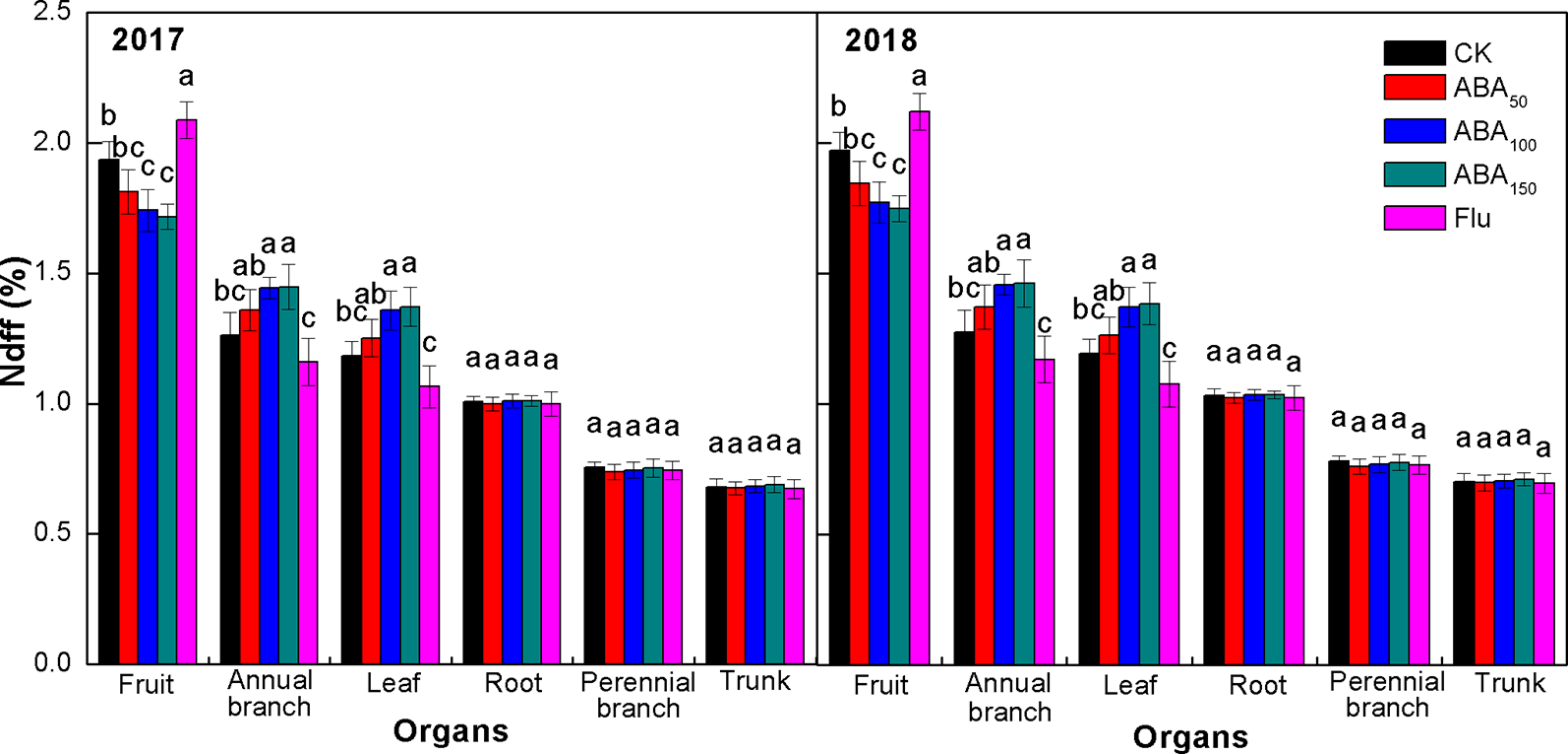
Effects of different treatments on Ndff at the fruit maturity stage in 2017 and 2018. The *vertical bar* indicates the standard deviation of three replications. *Different letters* indicate statistically significant differences (*P* < 0.05).

Ndff refers to the contribution rate of ^15^N absorbed from fertilizer and distributed by plant organs relative to the total nitrogen of plant organs and reflects the ability of plant organs to absorb and regulate ^15^N fertilizer. As presented in **Figure 4**, the Ndff values of organs subjected to different treatments were consistent at the fruit maturity stage in both years. The Ndff values of all treatments were fruit > annual branch > leaf > root > perennial branch > trunk (**Figure 4**). Compared with the control, exogenous ABA treatment decreased the Ndff value of fruits and increased the Ndff value of annual branches and leaves, while the Flu treatment significantly increased the Ndff value of fruits and significantly decreased the Ndff value of annual branches and leaves. The effects of different treatments on the Ndff values of storage organs (roots, perennial branches, and trunk) were not significant. The results showed that fruit had the strongest ability to absorb and regulate ^15^N at the fruit maturity stage. The ^15^N absorbed by trees was mainly distributed to fruits, and the annual branches and leaves also had strong competitiveness. Exogenous ABA treatment reduced the ability of fruit to absorb ^15^N and improved the ability of vegetative organs (annual branches and leaves) to absorb ^15^N. Fluridone treatment significantly improved the ability of fruit to absorb ^15^N and reduced the ability of vegetative organs to absorb ^15^N in both years.

**Figure 5:**
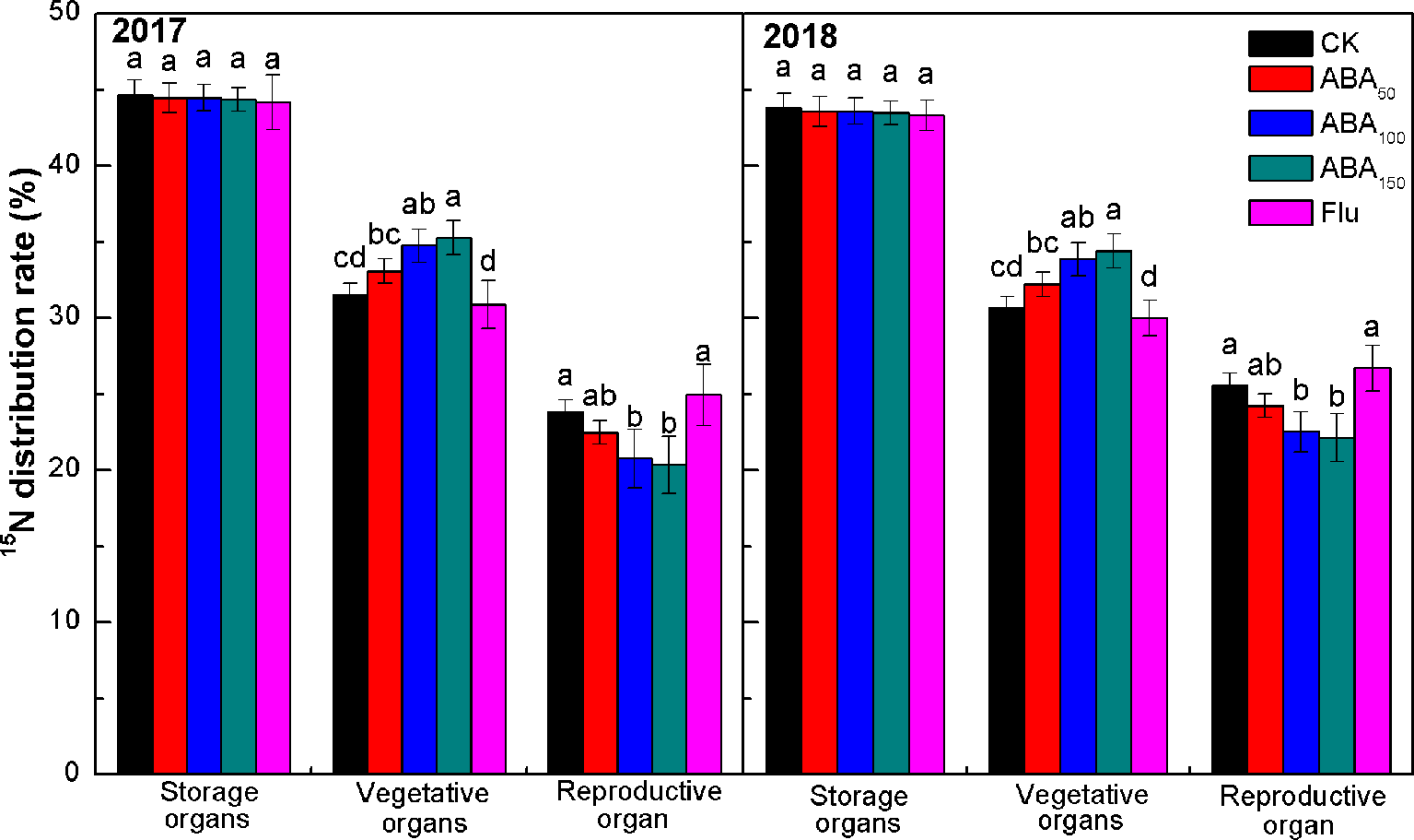
Effects of different treatments on the ^15^N distribution rate at the fruit maturity stage in 2017 and 2018 (^15^N distribution rate refers to the ratio of ^15^N absorbed by each organ from fertilizer to ^15^N absorbed by the plant from fertilizer). The *vertical bar* indicates the standard deviation of three replications. *Different letters* indicate statistically significant differences (*P* < 0.05).

The percentage of ^15^N in each organ to the total ^15^N in the whole plant reflects the distribution of N fertilizer in the tree and the migration of N fertilizer among the organs. As presented in **Figure 5**, the ^15^N distribution rate in different organs was consistent at the fruit maturity stage in 2017 and 2018. The value observed in the storage organs (perennial branches, roots, and trunk) was the highest, followed by the vegetative organs (annual branches and leaves) and then the reproductive organ (fruits). No treatment had a significant effect on the ^15^N distribution rate of storage organs. The ^15^N distribution rate of vegetative organs increased with the increase in exogenous ABA concentration, while the ^15^N distribution rate of reproductive organ decreased with the increase in exogenous ABA concentration. In 2017 and 2018, the ^15^N distribution rate of vegetative organs treated with 150 mg/L ABA was 11.91% and 12.25% higher than that of the control, and the ^15^N distribution rate of reproductive organ was 14.54% and 13.54% lower, respectively, than that of control. The ^15^N distribution rate of vegetative organs treated with fluridone was lower than that of the control, while the ^15^N distribution rate of reproductive organ was higher than that of the control. The results showed that exogenous ABA treatment reduced nitrogen migration from vegetative organs to fruits (**Figure 5**).

### Correlation Coefficient Among Exogenous ABA and Fruit-Related Indicators

As presented in **Supplementary Table S1**, the soluble sugar content increased first and then decreased with the increase in exogenous ABA concentration. The soluble sugar content in the 100-mg/L ABA treatment reached the highest level, which was 3.58% and 3.73% higher than that of the control in 2017 and 2018, respectively, while the soluble sugar content of the fluridone treatment was lower than that of the control.

With the increase of exogenous ABA concentration, ^15^N accumulation in fruit decreased, ^13^C accumulation in fruit increased first and then decreased, and the trend was consistent in both years (**Supplementary Table S2**).

As presented in **Table 3**, exogenous ABA treatment was positively correlated with the anthocyanin content in the peel (*r* = 0.6420, *P* > 0.05), soluble sugar content (*r* = 0.6595, *P* > 0.05), and ^13^C accumulation in fruits (*r* = 0.6978, *P* > 0.05) and was significantly and negatively correlated with ^15^N accumulation in fruits (*r* = −0.9935, *P* ≤ 0.01). The anthocyanin content in the peel was significantly and positively correlated with the soluble sugar content (*r* = 0.9941, *P* ≤ 0.01) and ^13^C accumulation in fruits (*r* = 0.9960, *P* ≤ 0.01) and was negatively correlated with the ^15^N accumulation in fruits (*r* = −0.7199, *P* > 0.05). The soluble sugar content was significantly and positively correlated with the ^13^C accumulation in fruits (*r* = 0.9872, *P* ≤ 0.05).

**Table 3:** Correlation coefficient among exogenous ABA and fruit-related indicators in both years.

	Exogenous ABA	Anthocyanin	Soluble sugar	^15^N accumulation in fruits	^13^C accumulation in fruits
Exogenous ABA	1.0000				
Anthocyanin	0.6420	1.0000			
Soluble sugar	0.6595	0.9941**	1.0000		
^15^N accumulation in fruits	−0.9935**	−0.7199	−0.7389	1.0000	
^13^C accumulation in fruits	0.6978	0.9960**	0.9872*	−0.7680	1.0000

The correlation coefficients were calculated by Pearson’s correlation based on the 2-year average. *P ≤ 0.05; **P ≤ 0.01.

## Discussion

### Effects of Exogenous ABA on the Synthesis of Endogenous ABA and Anthocyanin in Fruits

As one of the five plant hormones, ABA plays an important role in fruit color formation and regulation of ripening and senescence (Setha et al., 2004; Jia et al., 2011; Leng et al., 2014). The biosynthesis of anthocyanin is regulated by ABA (Oh et al., 2018). Exogenous ABA treatment can significantly increase the content of endogenous ABA in fruits and induce the upregulation of genes related to the anthocyanin–phenylpropane metabolic pathway and flavonoid pathway (Deluc et al., 2008; Gagné et al., 2011; Enoki et al., 2017). Exogenous ABA can promote the biosynthesis of anthocyanins in sweet cherry (Tijero et al., 2016), strawberry (Li et al., 2014; Li et al., 2015), grape (Hara et al., 2004; Koyama et al., 2014), and litchi (Wang et al., 2007; Hu et al., 2017) and can regulate the composition and content of pigments during the fruit-ripening period (Jiang and Joyce, 2003; Hara et al., 2004; Singh et al., 2014). In this study, 2 years of experiments showed that the ABA synthesis and receptor-related gene expression in the peel and flesh could be increased by exogenous ABA treatment, and the endogenous ABA content in the peel and flesh increased with the increase in the exogenous ABA concentration. The expression levels of anthocyanin synthesis genes (*MdCHS*, *MdCHI*, *MdF3H*, *MdDFR*, and *MdUFGT*) and transcription factors (*MdMYB1* and *MdbZIP44*) in the peel were significantly upregulated by ABA treatment at an appropriate concentration, and the anthocyanin content in the peel increased. The content of anthocyanin in the 150-mg/L ABA treatment was lower than that in the 100-mg/L ABA treatment, which indicated that exogenous ABA had a dose effect on the regulation of anthocyanin synthesis (**Table 2**). The correlation analysis showed that there was a positive correlation between the anthocyanin content in the peel and the exogenous ABA content, but the difference was not significant (**Table 3**). Unlike the results of our study, Sun et al. (2017) found that exogenous ABA inhibited anthocyanin synthesis in red-fleshed apple callus. Through gene expression analysis, they found that ABA inhibited the expression of anthocyanin structural genes, thereby inhibiting anthocyanin synthesis. This may be related to the different sensitivity of different plant materials to ABA, and the specific mechanism remains to be further studied.

### Exogenous ABA Regulates Anthocyanin Synthesis by Affecting Carbon–Nitrogen Nutrient and Sugar Accumulation in Fruits

Carbon and nitrogen metabolism is the most basic metabolic process in fruit growth and development. The degree of coordination of carbon and nitrogen metabolism and their transformation directly or indirectly affects fruit quality. The late growth stage of apple is the key period for fruit to convert from nitrogen nutrient to carbon nutrient. However, the high temperature and rainy weather at this stage lead to a large supply of soil nitrogen, resulting in vigorous nitrogen metabolism in the tree. The nitrogen content of fruit treated with control in this experiment is 2.86 g/kg, which is higher than the optimal nitrogen content of the high-quality apple fruit (Zhang et al., 2017). The high nitrogen content of the fruit prevents the formation of peel color (Kühn et al., 2011). Therefore, coordinating carbon–nitrogen nutrient through exogenous substances is beneficial to improve apple color.

There is evidence that ABA plays an important role in the unloading of photosynthetic products to fruits. For example, ABA promoted ^14^C transport from the wheat stem to the grain, and the ABA content was positively correlated with the ^14^C transport rate from the stem to the grain (Yang et al., 2010). Previous studies by our team found that exogenous ABA could promote the transport of photosynthates to fruits by enhancing the strength of the apple fruit bank (Sha et al., 2019). ABA treatment also enhanced the absorption of sugar by vacuoles in apple flesh (Yamaki and Asakura, 1991) and increased the sugar content in citrus fruit (Wang et al., 2016). Sugar plays multiple roles in anthocyanin synthesis, e.g., as an energy source and in osmotic regulation. Sugar is not only a precursor of anthocyanin synthesis but also a signal substance regulating anthocyanin synthesis (Smeekens, 2000; Solfanelli et al., 2006). ABA modulates the sucrose-induced expression of anthocyanin biosynthetic genes in *Arabidopsis* (Loreti et al., 2008) and regulates color formation by regulating anthocyanin biosynthesis and sugar accumulation (Kumar et al., 2014; Wang et al., 2015). In 2017 and 2018, our ^13^C and ^15^N double isotope labeling technology results showed that exogenous ABA treatment could improve the competitive ability of fruit with respect to ^13^C, increase the distribution of ^13^C from vegetative organs (leaves and annual branches) to fruit, reduce the capacity to absorb and regulate of fruit with respect to ^15^N, and reduce the amount of ^15^N transferred from vegetative organs to fruit. Inhibition of endogenous ABA synthesis (fluridone treatment) showed the opposite pattern, suggesting that ABA played an important role in regulating the carbon–nitrogen balance of fruits (**Figures 3**, **4**, **5**). The correlation analysis showed that exogenous ABA treatment was positively correlated with fruit ^13^C accumulation, the soluble sugar content, and the anthocyanin content and was significantly and negatively correlated with fruit ^15^N accumulation. The anthocyanin content in the peel was significantly and positively correlated with fruit ^13^C accumulation and the soluble sugar content and was negatively correlated with fruit ^15^N accumulation (**Table 3**). Exogenous ABA can coordinate carbon–nitrogen nutrient in the late stage of apple fruit development, reduce the accumulation of fruit nitrogen, increase the accumulation of fruit carbon, increase the sugar content of fruit, and promote anthocyanin synthesis by providing substrates or through sugar signaling mechanisms, thereby promoting apple coloration. However, the ^13^C accumulation in the treatment with a high concentration of ABA (150 mg/L ABA) was lower than that in the 100-mg/L ABA treatment (**Supplementary Table S2**). The reason may be that the 150-mg/L ABA treatment had a strong inhibitory effect on the nitrogen metabolism of fruits, resulting in insufficient precursors of carbon metabolism, thus affecting anthocyanin synthesis in the peel. The effect of exogenous ABA on nitrogen accumulation in fruit remains to be further studied.

## Conclusion

Exogenous ABA treatment upregulated the expression of ABA synthesis-related genes and increased the content of endogenous ABA in apple fruits. The accumulation of endogenous ABA triggered signal transduction and the regulation of ABA downstream signaling pathways and further stimulated the expression of anthocyanin synthesis genes to induce anthocyanin synthesis in the apple peel. On the other hand, exogenous ABA regulated the carbon–nitrogen balance of apple fruits, increased the sugar content of apple fruit, and promoted anthocyanin synthesis by providing substrates or through sugar signaling mechanisms. Comprehensive analysis showed that the application of 100 mg/L ABA to fruit at the late stage of apple development (135 days after blooming) could effectively improve the problem of poor coloration caused by high fruit nitrogen.

## Data Availability Statement

All datasets generated for this study are included in the article/**Supplementary Material**.

## Author Contributions

FW, JS, QC, ZZ, SG, and YJ conceived and designed the experiments. FW, SG, and YJ performed the experiments. FW, JS, QC, and ZZ wrote the manuscript. FW, JS, QC, XX, and ZZ analyzed the data. All authors have read and approved the final version of the manuscript.

## Funding

This work was supported by the Special Fund for the National Key R&D Program of China (2016YFD0201100), National Natural Science Foundation of China (31501713), China Agriculture Research System (CARS-27), and Taishan Scholar Assistance Program from Shandong Provincial Government.

## Conflict of Interest

The authors declare that the research was conducted in the absence of any commercial or financial relationships that could be construed as a potential conflict of interest.
