# Supplementary material for: Exogenous Abscisic Acid Regulates Distribution of 13C and 15N and Anthocyanin Synthesis in ‘Red Fuji’ Apple Fruit Under High Nitrogen Supply
Source: Front Plant Sci. 2020 Jan 24;10:1738. doi: 10.3389/fpls.2019.01738 (PMC6997889; doi:10.3389/fpls.2019.01738)
Supplement: Supplementary file 1 [file DataSheet_1.docx]

**Supplementary Table S1** **|** Effects of different treatments on the soluble sugar contents of fruit in 2017 and 2018.

| Year | Treatment | Soluble sugar (%) |
| --- | --- | --- |
| 2017 | CK | 13.96±0.40ab |
|  | ABA_50_ | 14.13±0.27ab |
|  | ABA_100_ | 14.46±0.50a |
|  | ABA_150_ | 14.20±0.32a |
|  | Flu | 13.43±0.37b |
| 2018 | CK | 14.22±0.42ab |
|  | ABA_50_ | 14.41±0.29ab |
|  | ABA_100_ | 14.75±0.53a |
|  | ABA_150_ | 14.48±0.34a |
|  | Flu | 13.67±0.39b |

Data are presented as the mean ± SD of three replicates. Different letters within a column indicate statistically significant differences between the means (*P* < 0.05).

**Supplementary Table S2 |** Effects of different treatments on the accumulation of ^15^N and ^13^C in fruits in 2017 and 2018.

| Year | Treatment | ^15^N accumulation in fruits (mg/plant) | ^13^C accumulation in fruits (mg/plant) |
| --- | --- | --- | --- |
| 2017 | CK | 114.83±3.23b | 148.86±11.28c |
|  | ABA_50_ | 109.87±4.95bc | 167.04±7.79b |
|  | ABA_100_ | 105.30±4.66bc | 186.06±13.45a |
|  | ABA_150_ | 104.42±5.06c | 169.99±5.68ab |
|  | Flu | 117.32±3.57a | 129.20±6.67d |
| 2018 | CK | 119.18±3.40ab | 156.47±11.89c |
|  | ABA_50_ | 115.36±8.20bc | 175.63±8.21b |
|  | ABA_100_ | 110.54±4.91bc | 195.69±14.18a |
|  | ABA_150_ | 108.50±5.33c | 178.75±5.99ab |
|  | Flu | 128.49±3.76a | 135.74±7.03d |

Data are presented as the mean ± SD of three replicates. Different letters within a column indicate statistically significant differences between the means (*P* < 0.05).
